# Modulation of light-driven arousal by LIM-homeodomain transcription factor Apterous in large PDF-positive lateral neurons of the *Drosophila* brain

**DOI:** 10.1038/srep37255

**Published:** 2016-11-17

**Authors:** Naoto Shimada, Show Inami, Shoma Sato, Toshihiro Kitamoto, Takaomi Sakai

**Affiliations:** 1 Department of Biological Sciences, Graduate school of science & engineering, Tokyo Metropolitan University, Tokyo, Japan; 2 Department of Anesthesia, University of Iowa, Iowa City, Iowa, United States of America; 3 Interdisciplinary Graduate Programs in Genetics and Neuroscience, University of Iowa, Iowa City, Iowa, United States of America

## Abstract

Apterous (Ap), the best studied LIM-homeodomain transcription factor in *Drosophila*, cooperates with the cofactor Chip (Chi) to regulate transcription of specific target genes. Although Ap regulates various developmental processes, its function in the adult brain remains unclear. Here, we report that Ap and Chi in the neurons expressing PDF, a neuropeptide, play important roles in proper sleep/wake regulation in adult flies. PDF-expressing neurons consist of two neuronal clusters: small ventral-lateral neurons (s-LNvs) acting as the circadian pacemaker and large ventral-lateral neurons (l-LNvs) regulating light-driven arousal. We identified that Ap localizes to the nuclei of s-LNvs and l-LNvs. In light-dark (LD) cycles, RNAi knockdown or the targeted expression of dominant-negative forms of Ap or Chi in PDF-expressing neurons or l-LNvs promoted arousal. In contrast, in constant darkness, knockdown of Ap in PDF-expressing neurons did not promote arousal, indicating that a reduced Ap function in PDF-expressing neurons promotes light-driven arousal. Furthermore, Ap expression in l-LNvs showed daily rhythms (peaking at midnight), which are generated by a direct light-dependent mechanism rather than by the endogenous clock. These results raise the possibility that the daily oscillation of Ap expression in l-LNvs may contribute to the buffering of light-driven arousal in wild-type flies.

The *Drosophila* LIM-homeodomain (LIM-HD) protein, Apterous (Ap), acts as a transcription factor, and it is evolutionarily conserved across species^1^. Similar to other LIM-HD proteins, Ap contains two LIM domains which mediate protein-protein interactions and a HD with a DNA-binding specificity (Fig. S1a)^1^. In the fruitfly *Drosophila melanogaster*, Ap plays important roles in wing development^2^,^3^,^4^, muscle development^5^,^6^, nervous system development^4^,^7^,^8^, neuropeptide expression^9^,^10^, and juvenile hormone production^11^. The interaction between Ap and its cofactor Chip (Chi) during wing development has been well-characterized as follows^12^: (1) a null mutation of *Chi* induces an *ap* mutant-like phenotype, (2) the Ap LIM domains interact with the LIM interaction domain (LID) of Chi, and Chi can homodimerize through the dimerization domain (DD) of Chi (Fig. S1a), (3) the multimeric Ap/Chi complexes regulate the transcription of Ap target genes (Fig. S1b). Although the transcriptional regulation through the Ap/Chi complex plays a key role in wing development, the roles of Ap and its cofactor in adult brain neurons is largely unknown.

The sleep-like state is widely conserved among animal species^13^,^14^, and *Drosophila* has been used in studies to clarify the genetic basis of sleep/wake regulation^15^,^16^. Genetic studies using *Drosophila* have identified several molecular components associated with sleep/wake regulation^14^,^17^, and the molecules identified in *Drosophila* are mostly shared by mammals^14^,^18^,^19^. The mouse Ap homolog Lhx9 is abundant in orexin-producing neurons in the hypothalamus, which regulates sleep/wake behaviors, and Lhx9 expression is essential for normal sleep behavior^20^. In *Drosophila*, *ap* is also expressed in restricted neuronal populations with wake-promoting effects, which are located in the ventral lateral region of the adult brain^21^. A neuropeptide, the pigment-dispersing factor (PDF), is released by central clock cells in the *Drosophila* brain^22^. PDF-expressing neurons (abbreviated as “PDF neurons”) consist of two clusters, small ventral-lateral neurons (s-LNvs) and large ventral-lateral neurons (l-LNvs)^22^,^23^. s-LNvs play an important role in the timing of the morning peak and circadian rhythms of locomotor activity in constant darkness (DD)^22^,^24^,^25^, whereas l-LNvs regulate light-driven arousal^26^,^27^,^28^. l-LNvs show a unique gene expression profile that differs from that of s-LNvs or non-PDF neurons. Microarray analysis revealed that the expression levels of 577 genes including *ap* are elevated in l-LNvs^21^. However, it remains unknown whether Ap expression in l-LNvs plays a crucial role in proper sleep/wake behaviors. This study revealed that Ap is expressed in some l-LNvs and s-LNvs in the adult brain, and indicated that Ap and Chi in l-LNvs are involved in sleep/wake regulation by buffering light-driven arousal.

## Results

### Ap is expressed in l-LNvs and s-LNvs in the adult brain

**Figure 1:**
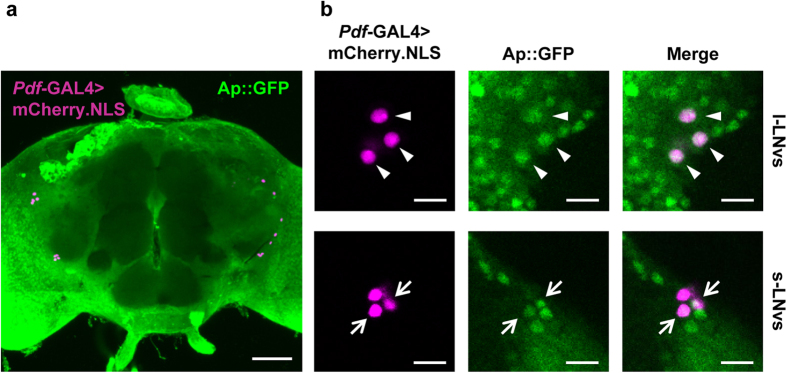
Ap expression in adult brain. Nuclei of PDF neurons were visualized using mCherry.NLS. *Pdf*-GAL4/*ap::GFP*; UAS-*mCherry.NLS*/+ flies were used. mCherry.NLS is shown in magenta, Ap::GFP is shown in green, and the overlap is shown in white. Adult brains were dissected at ZT18 after the flies were entrained to more than 3 LD cycles. (**a**) Stacked confocal image showing a front view of the adult brain. A scale bar represents 50 μm. (**b**) Confocal section image at the level of PDF neurons of the adult brain. Scale bars represent 10 μm. Triangles, l-LNvs; arrows, s-LNvs.

To examine whether Ap is expressed in PDF neurons, we used *ap::GFP* knock-in flies, which express a GFP reporter in a pattern consistent with endogenous Ap expression^29^. We observed the colocalization of Ap::GFP and the nucleus-targeted mCherry reporter for PDF neurons in *Pdf*-GAL4/*ap::GFP*; UAS-*mCherry.NLS*/+ flies, and confirmed that Ap localizes to the nuclei of PDF neurons including l-LNvs and s-LNvs (Fig. 1). Among l-LNvs and s-LNvs, 3–5 cells with mCherry.NLS signals were detected in each brain hemisphere [l-LNvs, 4 ± 0.2 (mean ± SEM) cells, *N* = 8; s-LNvs, 3.6 ± 0.2 cells, *N* = 8], and among them, 2–4 cells were GFP-positive [l-LNvs, 3 ± 0.4 cells, *N* = 8; s-LNvs, 2.4 ± 0.4 cells, *N* = 8].

### Targeted expression of *ap* RNAi in PDF neurons promotes arousal under LD conditions

**Figure 2:**
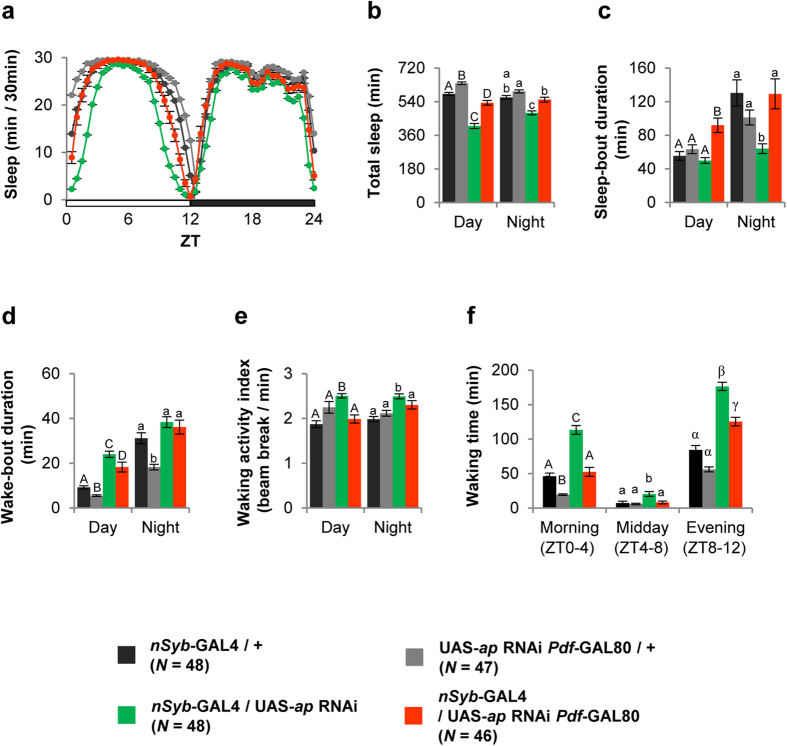
Pan-neural knockdown of *ap* promotes arousal under LD cycles. We generated transgenic flies (UAS-*ap* RNAi *Pdf*-GAL80) with both the UAS-*ap* RNAi and *Pdf*-GAL80 constructs in the second chromosome. Black circles and bars, *nSyb*-GAL4/+; gray circles and bars, UAS-*ap* RNAi *Pdf*-GAL80/+; green circles and bars, *nSyb*-GAL4/UAS-*ap* RNAi; orange circles and bars, *nSyb*-GAL4/UAS-*ap* RNAi *Pdf*-GAL80. All sleep/wake parameters (daily sleep pattern, total sleep amount, sleep-bout duration, wake-bout duration, waking time, and waking activity index) were analyzed using the data averaged over 3 days of LD. Error bars show S.E.M. in each figure. Bars with the same letter indicate values that are not significantly different (*P* > 0.05). (**a**) Daily sleep patterns of control and experimental flies. (**b**) Total sleep amount during day and night. (**c**) Sleep-bout durations during day and night. (**d**) Wake-bout durations during day and night. (**e**) Waking activity indices during day and night. (**f**) Waking times in the morning (ZT0–4), midday (ZT4–8), and evening (ZT8–12).

First, we examined whether *ap* expression is required in neurons for the expression of the proper sleep/wake phenotype. We knocked down *ap* in neurons by expressing *ap* RNAi using the pan-neuronal driver *nSyb*-GAL4 and analyzed sleep/wake behaviors under LD conditions. Pan-neuronal *ap* knockdown reduced the amount of sleep during both the day and night (Fig. 2a,b, green circles and bars). However, parameters for daytime and nighttime sleep are differentially affected by *ap* knockdown. For example, compared with control flies (Fig. 2c, black and gray bars), pan-neuronal knockdown of *ap* expression decreased sleep-bout duration but did not have a significant effect on wake-bout duration in the night (Fig. 2c,d, green bars). In contrast, *ap* knockdown increased wake-bout duration but kept sleep-bout duration unchanged during the day (Fig. 2c,d, green bars). These results indicate that pan-neuronal *ap* knockdown adversely affects sleep initiation during the day, while it disrupts sleep maintenance during the night. The waking activity index was slightly increased by *ap* knockdown during both the day and night (Fig. 2e, green bars). As seen in Fig. 2a, the sleep-suppressing effect of pan-neuronal *ap* knockdown was most significant during the daytime near dawn and dusk. Indeed, when the total waking time in the morning [Zeitgeber time (ZT) 0–4], midday (ZT4–8), and evening (ZT8–12) was compared between *ap*-knockdown flies and controls, differences were most obvious in the morning and evening (Fig. 2f).

To examine whether neuron-specific knockdown of *ap* affects circadian rhythms, we measured the locomotor activity of the *nSyb*-GAL4/UAS-*ap* RNAi flies for 10 days in DD and calculated the percentage of the flies showing rhythmic locomotor activity. Similarly to the control flies, *nSyb*-GAL4/UAS-*ap* RNAi flies showed rhythmic locomotor activity in DD (Fig. S2), indicating that *ap* knockdown does not affect circadian rhythms of locomotor activity.

Next, we examined the significance of PDF neurons in the wake-promoting effect of pan-neuronal *ap* knockdown by specifically abolishing GAL4 activity in PDF neurons using *Pdf*-GAL80^24^. The effect of pan-neuronal knockdown of *ap* was considerably mitigated when *Pdf*-GAL80 was included (Fig. 2a–f, orange circles and bars). This result suggests that Ap expression in PDF neurons is required for proper sleep/wake regulation, while Ap-positive non-PDF neurons also play a role. In all subsequent experiments, we focus on the total sleep amount (day and night) and waking time (morning, midday, and evening), because these sleep/wake parameters are most significantly affected by *ap* knockdown in a PDF neuron-dependent manner.

**Figure 3:**
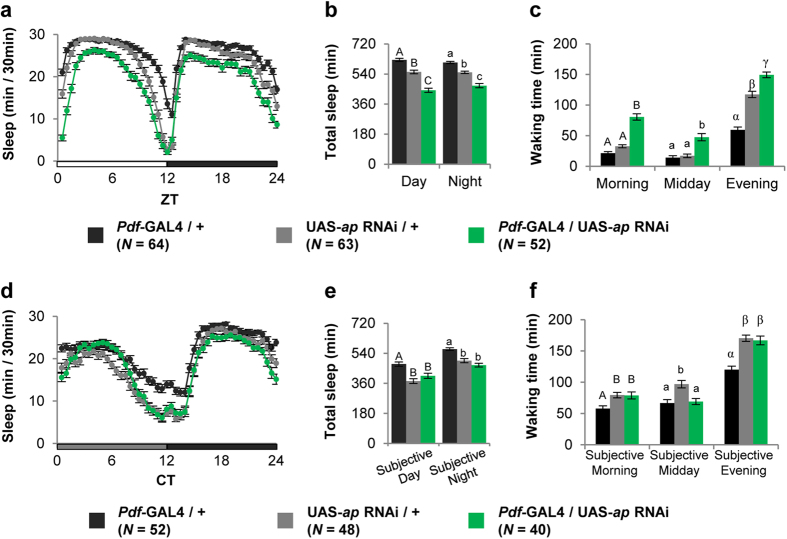
PDF neuron-specific knockdown of *ap* promotes arousal in LD cycles. Sleep/wake parameters were analyzed using the data averaged over 3 days of LD or DD. Error bars show S.E.M. in each figure. Bars with the same letter indicate values that are not significantly different (*P* > 0.05). (**a** and **d**) Daily sleep patterns of control and experimental flies. (**b** and **e**) Total sleep amount during day and night. (**c** and **f**) Waking times in the morning (ZT0–4 or CT0–4), midday (ZT4–8 or CT4–8), and evening (ZT8–12 or CT8–12). (**a–c**) Sleep was measured for 3 days in LD cycles after the flies were entrained to 3 LD cycles. (**d–f**) Sleep was measured in DD after the flies were entrained to 3 LD cycles.

The significance of Ap expression in PDF neurons was directly accessed by *ap* knockdown in a PDF neuron-specific manner using *Pdf*-GAL4 in combination with UAS-*ap* RNAi. Under LD conditions, the targeted expression of *ap* RNAi to PDF neurons also induced the characteristic sleep/wake phenotype that was observed for pan-neuronal *ap* knockdown. The amount of sleep during the day and night was reduced (Fig. 3a,b), and the total waking time in the morning, midday and evening increased (Fig. 3c). To confirm that the observed effects depend on *ap* knockdown, we examined *Pdf*-GAL4/UAS-*dOrk1Δ NC* flies as negative controls. dOrk1ΔNC is a nonfunctional isoform containing a K^+^ channel-inhibiting mutation; and it is known that induction of this isoform in specific neurons does not affect neural activity^30^. As we expected, dOrk1ΔNC expression in PDF neurons affected neither the sleep amount nor waking time (Fig. S3).

Genetic ablation of l-LNvs increases the amount of sleep in LD cycles, but this phenotype disappears after the transfer of flies from LD to DD conditions^28^. In addition, excitation of l-LNvs promotes arousal^26^,^28^. Thus, PDF-producing l-LNvs play a key role in light-driven arousal. To test whether the high-arousal phenotype in *ap-*knockdown flies is also light-dependent, we examined *Pdf*-GAL4/UAS-*ap* RNAi flies for their sleep/wake phenotype in DD. Compared with control flies, no significant differences were detected in sleep amount during the day and night or waking time in the morning [Circadian time (CT) 0–4], midday (CT4–8) and evening (CT8–12) (Fig. 3d–f), indicating that the wake-promoting effect induced by *ap* knockdown in PDF neurons is light-dependent. These results revealed that endogenous Ap in PDF neurons buffers light-driven arousal in wild-type flies.

### Targeted expression of truncated forms of Ap in PDF neurons promotes arousal

**Figure 4:**
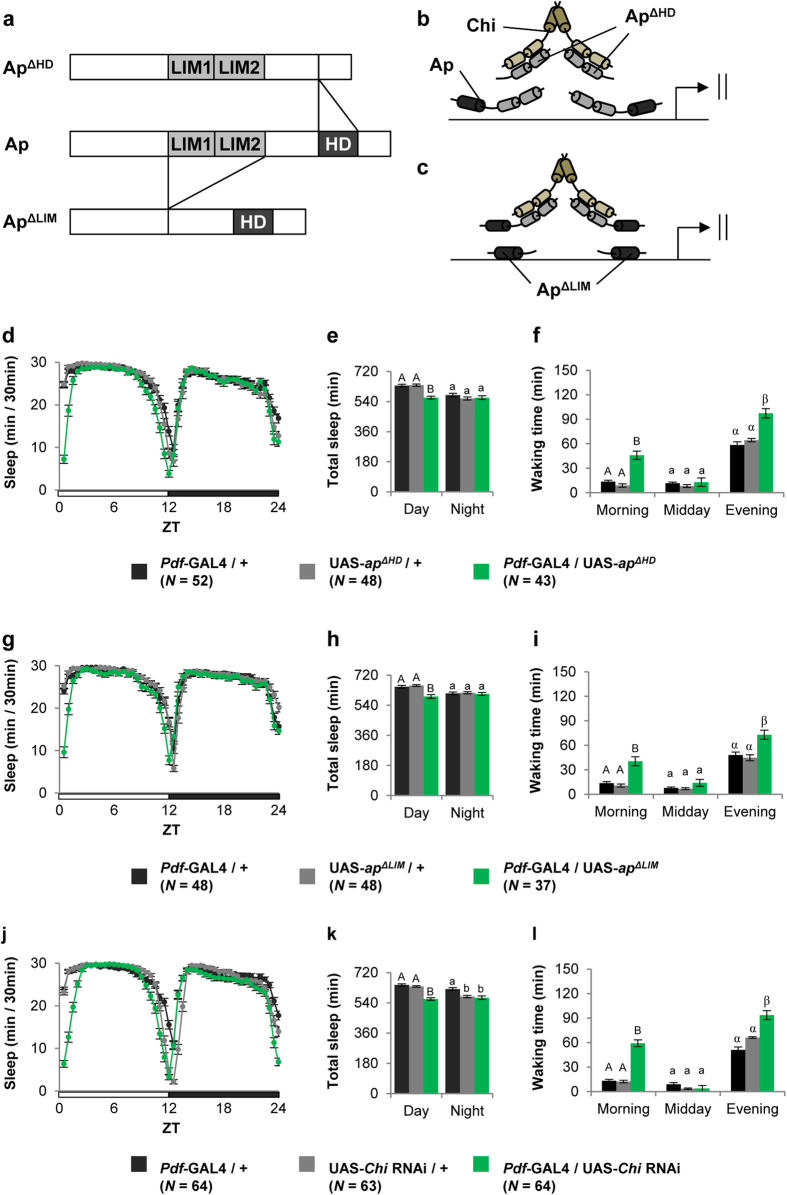
Targeted expression of truncated forms of Ap and *Chi* RNAi in PDF neurons promotes arousal. Sleep/wake parameters were analyzed using the data averaged over 3 days of LD. Error bars show S.E.M. in each figure. Bars with the same letter indicate values that are not significantly different (*P* > 0.05). (**a**) Schematic representation of wild-type and truncated forms of AP. (**b** and **c**) Possible model of dysfunction of Ap/Chi through expression of Ap^ΔLIM^ and Ap^ΔHD^. Sleep/wake parameters obtained using (**d**–**f**) *Pdf*-GAL4/UAS-*ap*^*ΔHD*^ flies, (**g**–**i**) *Pdf*-GAL4/UAS-*ap*^*ΔLIM*^ flies, and (**j**–**l**) *Pdf*-GAL4/UAS-*Chi* RNAi flies. (**d**,**g** and **j**) Daily sleep patterns of control and experimental flies. (**e**,**h** and **k**) Total sleep amount during day and night. (**f**, **i** and **l**) Waking times in the morning (ZT0–4), midday (ZT4–8), and evening (ZT8–12).

To further examine how disruption of the Ap function affects sleep/wake behaviors, we used two truncated forms of Ap (Ap^ΔLIM^ and Ap^ΔHD^; Fig. 4a). They are expected to act as a dominant negative protein and inhibit the transcriptional activity of the Ap/Chi complex because (1) Ap^ΔHD^ lacking a HD can reduce the amount of the functional Ap/Chi complex by sequestering endogenous Chi (Fig. 4b), and (2) Ap^ΔLIM^ lacking two LIM domains can interfere with the DNA binding of the Ap/Chi complex (Fig. 4c). In fact, Ap^ΔHD^ was shown to induce the dominant negative effect during wing development^4^. Here, we generated two transgenic lines, UAS-*ap*^*ΔHD*^ and UAS-*ap*^*ΔLIM*^, and examined the effects of Ap^ΔHD^ and Ap^ΔLIM^ on sleep/wake behaviors. PDF neuron-specific expression of Ap^ΔHD^ induced a significant decrease in sleep amount only during the day (Fig. 4d,e). As was observed in *Pdf*-GAL4/UAS-*ap* RNAi flies, morning and evening waking times increased (Fig. 4f). Similar dominant-negative effects on the sleep/wake phenotype were detected when Ap^ΔLIM^ was expressed in PDF neurons (Fig. 4g–i). These results further confirmed our finding that disruption of the Ap function promotes arousal during the day.

### Targeted expression of *Chi* RNAi and truncated forms of Chi in PDF neurons promotes arousal

Considering the dominant negative effects of Ap^ΔHD^ and Ap^ΔLIM^ in the sleep/wake phenotype, it is possible that the transcriptional activity of Ap/Chi is crucial to arousal regulation during the day. To investigate this possibility, we first examined whether the knockdown of *Chi* in PDF neurons also affects sleep/wake behaviors. During the day, *Pdf*-GAL4/UAS-*Chi* RNAi flies showed reduced sleep amount and lengthened waking time in the morning and evening (Fig. 4j–l). Next we used two dominant-negative forms of Chi (Chi^ΔLID^ and Chi^ΔDD^; Fig. S4). The transcriptional activity of Ap/Chi is expected to be inhibited by the induction of these truncated forms because they can interfere with the formation of the functional Ap/Chi tetramer. Actually, expression of Chi^ΔLID^ or Chi^ΔDD^ induces the dominant negative effects in wing development or posteclosion behavior requiring Chi functions^12^,^31^. As was observed in *Pdf*-GAL4/UAS-*Chi* RNAi flies, the targeted expression of Chi^ΔLID^ in PDF neurons reduced sleep amount during the day and increased waking time in the morning and evening (Fig. S5a–c). The expression of Chi^ΔDD^ induced weak but significant reduction in the amount of daytime sleep and lengthened the morning waking time (Fig. S5d–f). Thus, disruption of the Chi function, as well as Ap dysfunction, also promotes arousal during the day.

### *ap* knockdown in l-LNvs promotes arousal

**Figure 5:**
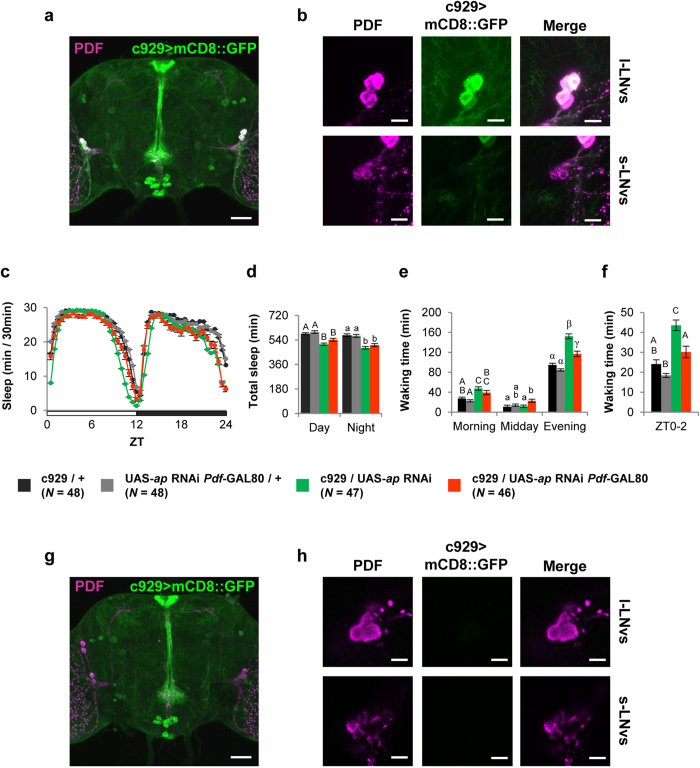
Knockdown of *ap* in l-LNvs promotes arousal. (**a** and **b**) c929-driven GFP (green) and PDF immunolabeling (magenta). (**a**) Stacked confocal image showing a front view of the adult brain. A scale bar represents 50 μm. (**b**) Confocal section image at the level of PDF neurons of the adult brain. Scale bars represent 10 μm. (**c**–**f**) c929/UAS-*ap* RNAi (green) and c929/UAS-*ap* RNAi *Pdf*-GAL80 (orange) flies were used. c929/+ (black) and UAS-*ap* RNAi *Pdf*-GAL80/+ (gray) flies were used as the control. (**c**) Daily sleep patterns of control and experimental flies. (**d**) Total sleep amount during day and night. (**e**) Waking times in the morning (ZT0–4), midday (ZT4–8), and evening (ZT8–12). (**f**) Waking time during the period between ZT0 and ZT2. (**g** and **h**) PDF immunolabeling (magenta) in c929/*Pdf*-GAL80; UAS-*mCD8::GFP*/+ flies. (**g**) Stacked confocal image showing a front view of the adult brain. A scale bar represents 50 μm. (**h**) Confocal section image at the level of PDF neurons of the adult brain. Scale bars represent 10 μm.

Ap is expressed in PDF-positive s-LNvs and l-LNvs (Fig. 1). Considering the effects of the PDF neuron-specific knockdown on daytime sleep and circadian rhythm, it is likely that Ap plays an important role in light-activated wake-promoting neurons, l-LNvs. To examine this possibility, we used a GAL4 line, c929, which expresses GAL4 in peptidergic neurons including l-LNvs^25^,^32^. As previously reported, GFP signals were detected in l-LNvs, but not in s-LNvs, in c929/UAS-*mCD8::GFP* flies (Fig. 5a,b). As was observed in *Pdf*-GAL4/UAS-*ap* RNAi flies, c929/UAS-*ap* RNAi flies also showed reduced sleep amount and increased waking time in the morning and evening (Fig. 5c–f, green circles and bars).

To examine the significance of Ap expression in l-LNvs in the regulation of daytime arousal, we used c929 in combination with *Pdf*-GAL80. We confirmed that the GFP signals in l-LNvs were abolished in c929 with *Pdf*-GAL80 (Fig. 5g,h). When c929 GAL4 activity was suppressed in PDF neurons expressing *Pdf*-GAL80, the total sleep and waking times in the morning and midday were not significantly altered in comparison with c929/UAS-*ap* RNAi flies (Fig. 5c–e, orange circles and bars), but the evening waking time significantly decreased (Fig. 5e). In addition, regarding the waking time between ZT0 and ZT2, no significant differences were detected between c929/UAS-*ap* RNAi *Pdf*-Gal80 and c929/+ control flies (Fig. 5f, orange bar). Thus, our results indicate that the enhanced-arousal phenotype in c929/UAS-*ap* RNAi flies is nearly rescued in c929/UAS-*ap* RNAi *Pdf*-GAL80 flies. We examined whether Ap::GFP is expressed in c929-positive non-PDF neurons using the c929/*ap::GFP*; UAS-IVS*-mCD8::RFP*/+ flies. We confirmed that Ap is expressed in several c929-positive cells located in the pars intercerebralis (PI), subesophageal zone (SEZ), and some neurons in the posterior brain region (Fig. S6), suggesting that these neurons may partially contribute to the enhanced-arousal phenotype in c929/UAS-*ap* RNAi flies.

We next used a GAL4 line, Mai179, which predominantly expresses GAL4 in s-LNvs and weakly expresses GAL4 in one or two cells of l-LNvs^33^. *ap* knockdown in Mai179-positive neurons did not affect sleep amount or waking time (Fig. S7, green circles and bars). Taken together, our results indicate that l-LNvs are predominantly responsible for the wake-promoting effect induced by *ap* knockdown.

### Targeted expression of *ap* RNAi in PDF neurons does not affect PDF expression, cell number, and number of PDF-positive varicosities

**Figure 6:**
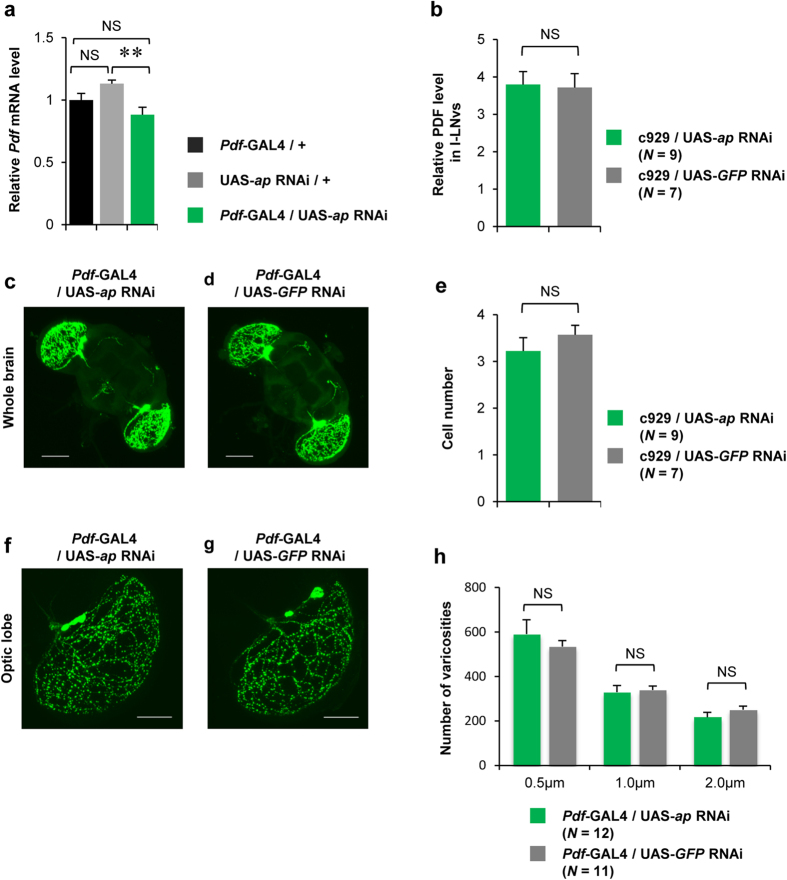
Knockdown of *ap* in l-LNvs does not affect PDF expression and number of PDF-releasing sites. (**a**) Real-time qRT-PCR analysis of *Pdf* mRNA expression level using *Pdf*-GAL4/+, UAS-*ap* RNAi/+, and *Pdf*-GAL4/UAS-*ap* RNAi males. Mean ± SEM values were calculated from five replicates. NS, not significant; **P < 0.01. (**b**) Intensity ratio of PDF signals in l-LNvs and s-LNvs (l-LNvs/s-LNvs). c929/UAS-*ap* RNAi (green bar) and c929/UAS-*GFP* RNAi (gray bar) flies were used. *N* = 7–8 in each bar. (**c** and **d**) PDF immunolabeling in the whole brain. Stacked confocal image showing a front view of the adult brain. A scale bar represents 50 μm. (**e**) Cell number in l-LNvs. (**f** and **g**) PDF immunolabeling in the optic lobe. Stacked confocal image of an optic lobe. Scale bars represent 20 μm. (**c** and **f**) *Pdf*-GAL4/UAS-*ap* RNAi flies were used. (**d** and **g**) *Pdf*-GAL4/UAS-*GFP* RNAi flies were used as the control. (**h**) Number of varicosities in l-LNvs. The number of spots of diameters (0.5, 1, and 2 μm) was counted for each diameter. *Pdf*-GAL4/UAS-*ap* RNAi (green bar) and *Pdf*-GAL4/UAS-*GFP* RNAi (gray bar) flies were used. 11–12 brains were used for each genotype.

Genetic ablation of l-LNvs inhibits light-driven arousal^28^ and excitation of l-LNvs promotes arousal^26^,^28^. In addition, *Pdf* null mutant flies show increased amount of daytime sleep^26^. These observations indicate that PDF production and release from l-LNvs promote light-driven arousal. We next examined whether Ap downregulates *Pdf* expression because some LIM-HD proteins act as a transcriptional repressor^1^,^34^. In qRT-PCR analyses, no significant difference was detected in *Pdf* mRNA expression levels between *Pdf*-GAL4/UAS-*ap* RNAi and control flies (*Pdf*-GAL4/+) (Fig. 6a). In addition, no significant difference was detected in PDF immunoreactivity in the cell bodies of l-LNvs between c929/UAS-*ap* RNAi and control flies (c929/ UAS-*GFP* RNAi) (Fig. 6b). Taken together, it is unlikely that PDF neuron-specific *ap* knockdown inhibits PDF expression. In addition, compared with control flies (*Pdf*-GAL4/UAS-*GFP* RNAi), no prominent structural defects in PDF-positive neurons was detected in *Pdf*-GAL4/UAS-*ap* RNAi flies (Fig. 6c,d), and *ap* knockdown did not affect the number of l-LNvs (Fig. 6e).

In *Drosophila*, l-LNvs project to the optic lobe and many PDF-positive varicosities, which are considered the sites of PDF release, exist in the optic lobe^35^. Thus, it is also possible that *ap* knockdown promotes arousal as a result of overproduction of PDF-releasing sites of l-LNvs. To address this possibility, we compared the number of PDF-positive varicosities in the optic lobe between *Pdf*-GAL4/UAS-*ap* RNAi and *Pdf*-GAL4/UAS-*GFP* RNAi flies. The mean number of PDF-positive varicosities of 0.5, 1, or 2 μm diameter was counted in each genotype. No significant difference was detected between *Pdf*-GAL4/UAS-*ap* RNAi and control flies (Fig. 6f–h), indicating that *ap* knockdown does not induce overproduction of PDF releasing sites of l-LNvs.

### Transient *ap* knockdown in l-LNvs promotes arousal in the morning

To determine whether transient *ap* knockdown in l-LNvs promotes arousal during the adult stage, we employed the TARGET system. Using UAS-*ap* RNAi/c929; *tub*-GAL80^ts^/+ flies, we performed temperature shift experiments (22 °C–30 °C–22 °C) as shown in Fig. S8a. First, we calculated daytime and nighttime sleep. In all genotypes, increase in the amount of daytime sleep and decrease in that of nighttime sleep were apparent at the restrictive temperature (Fig. S8b, L5; S8c, D4 and D5), indicating that the temperature shift itself modifies sleep/wake behaviors regardless of the genotype. Here, we calculated waking index (see Supplementary Material and Methods) to estimate the efficacy of transient *ap* knockdown for sleep/wake behaviors. In the morning (ZT0–4), midday (ZT4–8), and evening (ZT8–12), the waking index was defined as the difference between the mean waking time of GAL4 control flies (c929/+) and the waking time of each individual in UAS-*ap* RNAi/c929; *tub*-GAL80^ts^/+ or UAS control (UAS-*ap* RNAi/+; *tub*-GAL80^ts^/+) flies. Finally, mean waking index was calculated. In UAS-*ap* RANi/c929; *tub*-GAL80^ts^/+ flies, the mean waking index in the morning at the restrictive temperature was significantly higher than that at the permissive temperature (Fig. S8d, green bars, L5), but not in control flies (Fig. S8d, gray bars). Unlike the mean waking index in the morning, those in the midday and evening did not increase after a temperature shift (Fig. S8e and f, green bars, L5). Thus, these results suggest that at least morning arousal is promoted by transient *ap* knockdown. In UAS-*ap* RANi/c929; *tub*-GAL80^ts^/+ and UAS control flies, the mean waking index in the evening at the restrictive temperature significantly decreased in comparison with that during the first permissive temperature exposure (Fig. S8f, gray and green bars, L4 and L5). Thus, this reduction is not due to transient *ap* knockdown. However, in UAS-*ap* RANi/c929; *tub*-GAL80^ts^/+ flies, the mean waking index in the evening during the second permissive temperature exposure (Fig. S8f, grenn bars, L6–L8) recovered to the mean waking index during the first permissive temperature exposure (Fig. S8f, grenn bars, L4), but not in UAS control flies (Fig. S8f, gray bars). Although the reason for this differential recovery remains unclarified, it may be due to the combined effects of the gradual recovery of *ap* expression in c929-positive cells, the impact of the temperature shift itself (i.e., the shift modifies the physiological state of flies), and the genetic background of UAS-*ap* RANi/c929; *tub*-GAL80^ts^/+ flies.

### Ap expression in l-LNvs shows daily rhythms in LD, but not in DD

**Figure 7:**
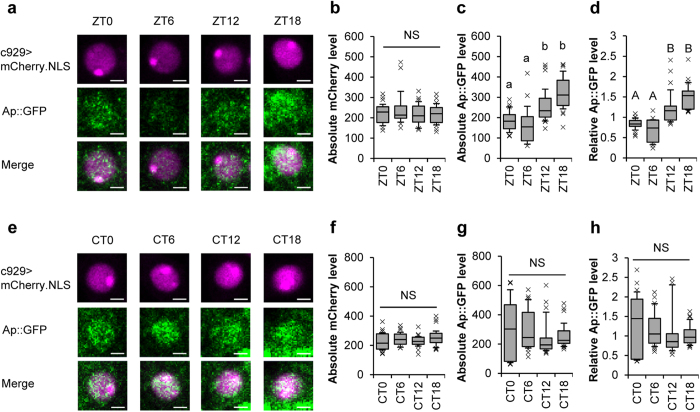
Ap is rhythmically expressed in l-LNvs under LD cycle conditions. (**a**) Ap::GFP expression in one of the l-LNvs at ZT0, 6, 12, and 18. c929/*ap::GFP*; UAS-*mCherry.NLS*/+ flies (6 days old) were used. Adult male brains were collected at 4 time points (ZT0, 6, 12, and 18) after the flies were entrained to 6 LD cycles. A scale bar represents 2 μm. (**b**) Absolute mCherry.NLS levels at ZT0, 6, 12, and 18. In l-LNvs, the c929-driven mCherry.NLS expression level remained nearly constant throughout the day. Thus, we used the mCherry.NLS as an internal standard. (**c**) Absolute Ap::GFP levels at ZT0, 6, 12, and 18. (**d**) Relative Ap::GFP levels at ZT0, 6, 12, and 18. (**b**–**d**) 8–10 brains were used at each time point. The total numbers of l-LNvs observed are as follows: *N* = 27 at ZT0, *N* = 15 at ZT6, *N* = 28 at ZT12, *N* = 23 at ZT18. (**e**) Ap expression in l-LNvs under DD. For measurement of Ap::GFP expression level in DD, adult male brains were collected at 4 time points (CT0, 6, 12, and 18) on the third day of DD after the flies were entrained to 3 LD cycles. A scale bar represents 2 μm. (**f**) Absolute mCherry.NLS levels at CT0, 6, 12, and 18. (**g**) Absolute Ap::GFP levels at CT0, 6, 12, and 18. (**h**) Relative Ap::GFP levels at CT0, 6, 12, and 18. (**f**–**h**) 9 brains were used at each time point. The total numbers of l-LNvs observed are as follows: *N* = 27 at CT0, *N* = 29 at CT6, *N* = 26 at CT12, *N* = 28 at CT18. (**b**–**d** and **f**–**h**) In each box plot, the box encompasses the interquartile range, a line is drawn at the median, and the vertical bars extend to the 10^th^ and 90^th^ percentiles. Crosses show outliers. Data sets with the same letter indicate that there are no significantly differences among them (*P* > 0.05). NS, not significant.

In the genome-wide expression analysis using all transcripts from the fly head, Claridge-Chang *et al.*^36^ have revealed that *ap* expression shows 24 h oscillation under LD conditions and the expression level of *ap* mRNA peaks detected in the middle of the night (around ZT16)^36^. Here, we examined whether Ap expression in l-LNvs also shows daily rhythms in LD cycles using c929/*ap::GFP*; UAS-*mCherry.NLS*/+ flies. The fluorescence intensity of Ap::GFP and mCherry.NLS in l-LNvs was measured at ZT0, ZT6, ZT12, and ZT18. Although the absolute mCherry.NLS fluorescence intensity level in l-LNvs did not change within 1 day (Fig. 7a,b), absolute and relative Ap::GFP levels increased during the night and peaked at ZT18 (Fig. 7c,d). To examine whether this oscillation is light-dependent, we next measured Ap::GFP levels in DD. As shown in Fig. 7e–h, clear oscillation of Ap::GFP expression was not detected in l-LNvs in DD (Fig. 7e–h). Taken together, we concluded that daily rhythms of Ap expression in l-LNvs is generated by a direct light-dependent mechanism not the circadian clock.

## Discussion

As shown in Fig. 1, Ap is expressed in many adult brain neurons. Nevertheless, no study on the roles of Ap in the adult brain has been conducted. The RNAi technology or dominant-negative transgenes of Ap can be used to identify brain-specific functions of Ap. In this study, we identified a novel role of the *Drosophila* LIM-HD protein Ap in PDF neurons. Previous studies revealed that activation of PDF neurons promotes arousal, whereas electrical silencing or genetic ablation of PDF neurons inhibits arousal^26^,^28^. In addition, a mutation of *Pdf* also inhibits arousal^26^. Thus, *Drosophila* arousal can be promoted or inhibited by genetic manipulations of PDF neurons. In this study, we identified that Ap localizes to the nuclei of PDF-positive l-LNvs and s-LNvs (Fig. 1). Targeted *ap* knockdown in PDF neurons or l-LNvs, but not in s-LNvs, enhanced arousal under LD conditions (Figs 3, 5, S6), indicating that Ap expression in l-LNvs buffers arousal in wild-type flies. Although the Ap function in s-LNvs remains unclarified in this study, our results support the idea that Ap-positive s-LNvs have little effect on light-driven arousal. Thus, our results indicate that the Ap function in l-LNvs differs from that in s-LNvs. PDF neuron-specific expression of Ap^ΔLIM^, Ap^ΔHD^, Chi^ΔLID^, and Chi^ΔDD^ increased waking time and consequently reduced sleep amount during the day (Figs 4, S5). All the truncated forms of Ap and Chi showed more or less dominant negative effects accompanied by the wake-promoting effect, indicating that Ap/Chi-dependent transcription in l-LNvs moderately buffers arousal during the day. Unlike in LD, *ap* knockdown in PDF neurons did not affect the sleep/wake phenotype in DD (Fig. 3). Taken together, our results reveal that the high-arousal phenotype induced by a reduced Ap function in PDF neurons is light-dependent and Ap expression in PDF neurons buffers light-driven arousal in wild-type flies.

*Drosophila* sleep is modulated by external signals (e.g., light and temperature) and internal signals (e.g., circadian clock, sleep pressure, and hunger)^16^,^37^,^38^. Since the induction of arousal is determined by the balance between external and internal impacts, neural mechanisms, which positively and negatively regulate arousal level, are required for keeping the suitable quality and/or quantity of sleep/wake behaviors. An electrophysiological study indicates that the firing of l-LNvs is induced by light inputs^39^,^40^,^41^, and the enhanced-sleep phenotype in LD induced by l-LNv-specific cell ablation disappears after the transfer of flies from LD to DD conditions^28^. Thus, it is considered that PDF-positive l-LNvs receive light signals and light-induced activation of these neurons triggers arousal. In contrast, Shang *et al.* have reported that the dopamine D2 receptor (D2R) buffers the effectiveness of dopamine-evoked cAMP responses in the l-LNvs in LD, but not in DD, leading to the downregulation of the wake-promoting effect of dopamine in a light-dependent manner^42^. Thus, l-LNvs can promote or buffer light-driven arousal depending on their molecular and physiological properties. In this study, we identified that endogenous Ap expression in l-LNvs buffers light-driven arousal. In addition, light-dependent oscillation of Ap expression was detected in l-LNvs and the maximum expression level of Ap was detected at the midnight (Fig. 7). Taken together, in wild-type flies, it is possible that the increased expression level of Ap at night leads to the characteristic physiological state required for the weakening of light-driven arousal. Although the detailed molecular functions of Ap in l-LNvs relevant to the weakening of light-driven arousal still remains unclarified, further studies associated with Ap-dependent gene expression profiling in l-LNvs will provide new insights into the molecular and neural mechanisms of light-driven arousal in *Drosophila*.

Although the loss of PDF production enhances sleep^26^, our results showed that *ap* knockdown does not have significant impacts on PDF expression (Fig. 6a,b) and the number of PDF-releasing sites (Fig. 6f–h). Thus, it seems unlikely that the increased PDF expression level or overproduction of PDF-releasing sites causes the wake-promoting effect induced by the knockdown of *ap*. Previous studies have revealed that homozygous mutations of *ap* affect various developmental processes, whereas heterozygous mutations of *ap* does not induce particular developmental defects in neurons^4^,^7^. In *Pdf*-GAL4/UAS-*ap* RNAi flies, anti-PDF antibody staining revealed that the number and morphology of PDF neurons seem to be intact (Fig. 6). In addition, transient *ap* knockdown was sufficient to promote morning arousal (Fig. S8). Taken together, high-arousal phenotype induced by *ap* knockdown does not simply results from the developmental effects of reduced AP function on l-LNvs. Unlike *ap-*knockdown flies with the high-arousal phenotype, Lhx9 knockout (KO) mice, in which more than 30% of orexin neurons are lost, exhibit the narcolepsy-like phenotype^20^. Thus, these two homologous transcription factors, *Drosophila* Ap and mouse Lhx9, may have distinct roles in wake-promoting neurons.

We confirmed that wake-promoting effect induced by pan-neuronal *ap* knockdown is partially rescued by *Pdf*-GAL80 (Fig. 2), suggesting that Ap in non-PDF neurons is also required for proper sleep/wake regulation. In *Drosophila* as well as other animal species, particular neurotransmitter systems play a key role in sleep/wake regulation^14^. For example, the involvement of dopaminergic, GABAergic, and peptidergic neurons are well characterized^14^,^26^,^43^,^44^,^45^,^46^. In particular, *Drosophila* sleep/wake behaviors is modified by several neuropeptides, including the short neuropeptide F, neuropeptide F, SIFamide, and Ion transport peptide^47^,^48^,^49^,^50^,^51^. Previous studies have revealed that Ap is necessary for expression of neuropeptides [FMRFamide and Leucokinin (Lk)] in the central nervous system^9^,^10^. Cavey *et al.* have reported that activating Lk-expressing neurons in the adult brain increases sleep amount in an Lk-receptor-dependent manner and Lk-expressing neurons regulate sleep levels by inhibiting Lk receptor-expressing neurons^52^. Although it remains unclear whether Ap-dependent Lk expression in adult brain regulates sleep amount, Ap-positive Lk neurons in the brain may also contribute to the Ap-dependent high-arousal phenotype in *Drosophila*.

The targeted expression of *Chi* RNAi and dominant negative transgenes of *ap* and *Chi* decreased the sleep amount only during the day (Fig. 4 and Fig. S5). This result was somewhat unexpected because the induction of *ap* RANi driven by *nSyb*-GAL4, *Pdf*-GAL4, and c929 decreased the sleep amount whether it was daytime or nighttime. Using a highly sensitive off-target search software (dsCheck)^53^, we confirmed that there is no significant off-target gene candidate toward *ap* RNAi sequences. Thus, it is unlikely that the sleep reduction during the night simply results from off-target effects of *ap* RNAi. Thus, it is possible that Ap in l-LNvs inhibit nighttime sleep in a Chi-independent manner. LIM-HD proteins can interact with different types of cofactor other than Chi-like LIM domain-binding proteins (LDB) and the complexes lead to transcriptional activation or repression^1^. Although the molecular mechanisms of Ap-dependent regulation of night sleep still remain unknown, at least our results suggest that the regulatory mechanisms of Ap-dependent sleep/wake during the night are different from those during the day.

## Materials and Methods

### Fly stocks

Fly stocks used for this study are as follows: wild-type *Drosophila melanogaster* Canton-S (CS), *ap::GFP* [Bloomington stock center (BS), *#*38423], *Pdf*-GAL4 (BS, #6900), *nSyb*-GAL4 (BS, #51941), c929 (BS, #25373), Mai179 (obtained from Dr. Orie T. Shafer, University of Michigan), *Pdf*-GAL80 (obtained from Leslie C. Griffith, Brandeis University), UAS-ap RNAi (NIG-fly, 8376R-1), UAS-*ap*^*ΔHD*^ (see next section), UAS-*ap*^*ΔLIM*^ (see next section), UAS-*Chi RNAi* (VDRC, 43934), UAS-*Chi*^*ΔLID*^ (obtained from Dr. Veronica Rodriguez, Tata Institute of Fundamental Research), UAS-*Chi*^*ΔDD*^ (obtained from Dr. Veronica Rodriguez), UAS-*dORK1ΔNC* (BS, #6587), UAS-*mCherry.NLS* (BS, #38424), UAS-IVS-*mCD8::RFP* (BS, #32218), and UAS-*GFP* RNAi (NIG-fly, GFP-IR-2). Flies were raised on glucose-yeast-cornmeal medium at 25.0 ± 0.5 °C in a 12-h light:12-h dark (LD) cycle. All lines except for UAS-*mCherry.NLS* and UAS-IVS-*mCD8::RFP* were outcrossed for at least six generations to *white* flies with the CS genetic background.

### Generation of UAS-*ap* ^*ΔHD*^ and UAS-*ap* ^*ΔLIM*^ transgenic flies

Full-length *ap* cDNA was isolated by RT-PCR using adult fly head RNA and two primers, 5′-GCGGCCGCCAAAATGGGCGTCTGCACCGAGGAGCGC-3′ and 5′TCTAGATTAGTCCAAGTTAAGTGGCGGTGTGC-3′.

The PCR product was digested with NotI and XbaI, and cloned into a pBluescript (pBS) II SK(+). Constructs of *ap*^*ΔHD*^ and *ap*^*ΔLIM*^ were generated by self-ligation of the PCR products amplified from the *ap* cDNA-containing pBS II SK(+) using two primer sets as follows: *ap*^*ΔHD*^ –forward, 5′-ATGATGAAGCAGGATGGCAGCGGC-3′; *ap*^*ΔHD*^ –reverse, 5′-CGACGAGGAGCTTAGGTGCGAGCC-3′; *ap*^*ΔLIM*^ –forward, 5′-GGGGATACCGCCTCATCCAGTATG-3′; *ap*^*ΔLIM*^ –reverse, 5′-GAGGTTGCGCGTTATTTTGCTATC-3′. The fragments lacking nucleotides 436–807 (*ap*^*ΔLIM*^) and 1096–1275 (*ap*^*ΔHD*^) of *ap* cDNA were obtained by restriction enzyme digestion using NotI and XbaI, and then they subcloned into the NotI/XbaI-digested pUAST attB vector^54^. Constructs of UAS-*ap*^*ΔHD*^ and UAS-*ap*^*ΔLIM*^ were injected into the eggs of PBac{y[+]-attP-9A}VK00005 (BS, #24862).

### Sleep analysis

Two- to three-day-old adult male flies were individually placed in a glass tube (5 mm × 65 mm) with fly food and monitored in a 12-h L:12-h D (LD) cycle (lights on at 8:00) at 25 °C. The locomotor activity of individual flies was analyzed using the DAM system (Trikinetics). Flies were acclimated in the glass tubes for 3 days in LD cycles before measurement of sleep. Locomotor activity data were collected at 1-min intervals for 3 days and analyzed with a Microsoft Excel-based program as described previously^55^. Sleep was defined as 5 min or more of behavioral inactivity, as previously described^56^. Total sleep amount, sleep- and wake- bout durations, waking activity, and waking time in the morning (ZT0–4 or CT0–4), midday (ZT4–8 or CT4–8) and evening (ZT8–12 or CT8–12) were analyzed for each 12-h period of LD or DD and averaged over 3 days for each condition. The waking activity was calculated by dividing the total activity counts during the length of the wake period during the day and night as reported previously^57^.

The Kolmogorov–Smirnov test was used to estimate whether the data are normally distributed. When the data were not distributed normally, we carried out the log transformation of the data. When the basic data or transformed data are distributed normally, one-way ANOVA followed by post-hoc analysis using Scheffe’s test was carried out for multiple pairwise comparisons. For multiple group analysis of the nonparametric data, we used nonparametric ANOVA (Kruskal–Wallis test) followed by the rank-sum test for multiple pairwise comparisons. The computer software IBM SPSS statistics22 (IBM Japan, Ltd.) was used for these tests.

### Behavioral rhythms

The flies were entrained to 12:12 LD cycles during their development, and 1-d-old single males were placed in glass tubes containing standard food, and their activity was monitored using the DAM system (Trikinetics). Infrared beam crosses in 30 min bins were recorded. We examined the effects of panneural knockdown of *ap* on circadian locomotor rhythms. Activity was monitored for 4–5 days of LD at 25 °C, followed by 10 days of DD at 25 °C. The circadian period and rhythmicity were estimated from the data of locomotor activity collected for 10 days of DD. Significant circadian rhythmicity was defined as the presence of a peak in periodogram power that extends above the significance line (*P* < 0.05) in chi-square analysis. Clocklab software (Actimetrics) was used to analyze the circadian period and rhythmicity.

### Real-time quantitative reverse transcription PCR (qRT-PCR)

Using TRizol (Invitrogen), total RNA was isolated from approximately 30 male fly heads of each genotype. cDNA was synthesized by a reverse transcription reaction using a QuantiTect Reverse Transcription Kit (QIAGEN). Real-time quantitative PCR was carried out using THUNDERBIRD SYBR qPCR Mix (TOYOBO) and a Chromo 4 Detector (MJ Research, Hercules, CA). Expression levels of *Pdf* mRNA were normalized by those of *rp49* mRNA. The average normalized *Pdf* mRNA expression levels in control flies was calculated using data from five independent assays. The ratio of normalized *Pdf* mRNA expression level in each experimental genotype to the average control value was calculated. The mean (± SEM) ratio was calculated for data from five independent assays. The primer sequences used for real-time PCR were as follows: *Pdf* –forward, 5′-ATCGGGATCTCCTCGACTGG-3′; *Pdf* –reverse, 5′-ATGGGCCCAAGGAGTTCTCG-3′; *rp49*–forward, 5′-AAGATCGTGAAGAAGCGCAC-3′; *rp49*–reverse, 5′-TGTGCACCAGGAACTTCTTG-3′.

### Immunohistochemistry

Adult male brains (over 5 days old) were fixed in PBS containing 4% formaldehyde for 45–60 min at room temperature. After three washes in PBST (0.1–0.2% Trion X-100 in PBS), they were blocked for 1 h in 1% normal goat serum in PBST and then incubated with a primary antibody. Next, they were incubated with a secondary antibody for 24 h at 4 °C after three washes in PBST.

For PDF staining, brains were stained with a mouse anti-PDF antibody (The Developmental Studies Hybridoma Bank at the University of Iowa, 1:2000), and Alexa Fluor 568 anti-mouse IgG (Invitrogen A11004) was used as the secondary antibody (1:1000). For GFP staining, brains were stained with a rabbit anti-GFP antibody (Invitrogen A11122, 1:200), and Alexa Fluor 488 anti-rabbit IgG (Invitrogen, A11008) was used as the secondary antibody (1:1000). Fluorescence was observed under a confocal microscope (Carl Zeiss LSM710 and Nikon C2).

### Quantitative analysis of PDF-positive cells or PDF immunoreactivity in l-LNvs

To examine whether *ap* knockdown in l-LNvs affects PDF-positive cell number or PDF immunoreactivity, c929/UAS-*ap* RNAi flies (6 days old) were used. c929/UAS-*GFP* RNAi flies were used as the control. The brain was dissected between ZT0 and ZT2. A confocal image stack of the brain hemisphere containing l-LNvs and s-LNvs was Z-projected into several sequential sections. Z-sections were collected at 0.53 μm intervals. We counted the number of PDF-positive cell bodies, and then the PDF immunoreactivity was quantified in a manually set region of interest (ROI) of the cell body region in each l-LNv and s-LNv using a histogram tool of ZEN 2010 software (Carl Zeiss). To compensate for the differences in fluorescence intensity between different ROIs, PDF immunoreactivity in l-LNvs was normalized to that in s-LNvs because c929 does not induce GAL4-dependent gene expression in s-LNvs. In all samples, the image data were acquired under identical conditions. The mean relative PDF immunoreactivity was calculated for each genotype. Student’s *t*-test was used for pair-wise comparison.

### Quantitative analysis of PDF-positive varicosities

Quantitative analysis of PDF-positive varicosities was conducted in the *Pdf*-GAL4/UAS-*ap* RNAi and control (*Pdf*-GAL4/UAS-*GFP* RNAi) flies. The brain was dissected between ZT0 and ZT2. A confocal image stack of one side of optic lobes was Z-projected (1.0 μm intervals). The PDF-positive varicosities on one side of the optic lobes of each brain were counted using the spot detection algorithm in Imaris software 7.1.0 (Bitplane). The diameters of spots were set at 0.5, 1, and 2 μm, and the number of spots was counted for each diameter. The mean number of varicosities was calculated for each genotype. Student’s *t*-test was used for pair-wise comparison.

### Quantitative analysis of Ap::GFP signal in l-LNvs

c929/*ap::GFP*; UAS-*mCherry.NLS*/+ flies (6-d-old) were used to measure Ap::GFP expression level in l-LNvs. For the measurement of Ap::GFP level in LD cycles, adult male brains were collected at 4 time points (ZT0, 6, 12, and 18) after the flies were entrained to 6 LD cycles. For the measurement of Ap::GFP level in DD, adult male brains were collected at 4 time points (CT0, 6, 12, and 18) on the third day of DD after the flies were entrained to 3 LD cycles. After extraction of the brains, they were stained with a rabbit anti-GFP antibody as described above. A confocal image stack of the brain hemisphere containing mCherry. NLS-positive- and Ap::GFP-positive-l-LNvs was Z-projected into 5–7 sequential sections (0.53 μm intervals). mCherry.NLS and Ap::GFP levels in l-LNvs were measured on the basis of mCherry or GFP fluorescence intensity determined using the colocalization tool of ZEN 2010 software (Carl Zeiss). Fluorescence intensity was measured in a manually set ROI of the mCherry.NLS-positive nuclear region in each l-LNv. To compensate for differences in fluorescence intensity between different ROI, Ap::GFP fluorescence intensity was normalized to the fluorescence intensity of mCherry.NLS. For all samples, their image data were acquired under identical conditions. Using the computer software BellCurve for Excel (Social Survey Research Information Co., Ltd.), nonparametric ANOVA (Kruskal–Wallis test) followed by post-hoc analysis using the Steel-Dwass test was carried out for multiple pairwise comparisons.

## Additional Information

**How to cite this article**: Shimada, N. *et al.* Modulation of light-driven arousal by LIM-homeodomain transcription factor Apterous in large PDF-positive lateral neurons of the *Drosophila* brain. *Sci. Rep.* **6**, 37255; doi: 10.1038/srep37255 (2016).

**Publisher’s note:** Springer Nature remains neutral with regard to jurisdictional claims in published maps and institutional affiliations.

## Supplementary Material

Supplementary Information
