# Supplementary material for: Modulation of light-driven arousal by LIM-homeodomain transcription factor Apterous in large PDF-positive lateral neurons of the Drosophila brain
Source: Sci Rep. 2016 Nov 17;6:37255. doi: 10.1038/srep37255 (PMC5112534; doi:10.1038/srep37255)
Supplement: Supplementary Information [file srep37255-s1.pdf]

Supplementary information

Modulation of light-driven arousal by LIM-homeodomain transcription factor Apterous in large PDF-positive lateral neurons of the *Drosophila* brain

Naoto Shimada[1*], Show Inami[1*], Shoma Sato[1], Toshihiro Kitamoto[2,3], and Takaomi Sakai[1]

[1]Department of Biological Sciences, Tokyo Metropolitan University, Tokyo, Japan,
[2]Department of Anesthesia, [3]Interdisciplinary Graduate Programs in Genetics and Neuroscience, University of Iowa, Iowa City, Iowa, United States of America.
*These authors contributed equally to this work.
Corresponding author, sakai-takaomi@tmu.ac.jp

Supplementary Materials and Methods

Transient *ap* knockdown in l-LNvs. For transient *ap* knockdown during the period of 1.5 days in the adult stage, c929/UAS-*ap* RNAi; *tub*-GAL80^{ts}/+ flies were used. c929/+ and UAS-*ap* RNAi/+; *tub*-GAL80^{ts}/+ flies were used as the control. These flies were kept at 22 ^{o}C, and three- to four-day-old adult male flies were individually placed in a glass tube. During the period between ZT0 on the 1st day and ZT 12 on the 4th day, individual flies were kept in a LD cycle at 22 ^{o}C, and then, they were kept in a LD cycle at 30 ^{o}C until ZT24 on the 5th day. After that, they were kept in a LD cycle at 22 ^{o}C until the 8th day. We calculated the waking index in individual flies as follows. First, the mean waking time of c929/+ control flies ($GAL4_{mean}$) was calculated in the morning (ZT0-4), midday (ZT4-8), and evening (ZT8-12) on each day, and then the difference between $GAL4_{mean}$ and the waking time of each individual in UAS-*ap* RNAi/c929; *tub*-GAL80^{ts}/+ and control (UAS-*ap* RNAi/+; *tub*-GAL80^{ts}/+) flies was calculated. Finally, for UAS-*ap* RNAi/c929; *tub*-GAL80^{ts}/+ and control (UAS-*ap* RNAi/+; *tub*-GAL80^{ts}/+) flies, their mean waking index was calculated.

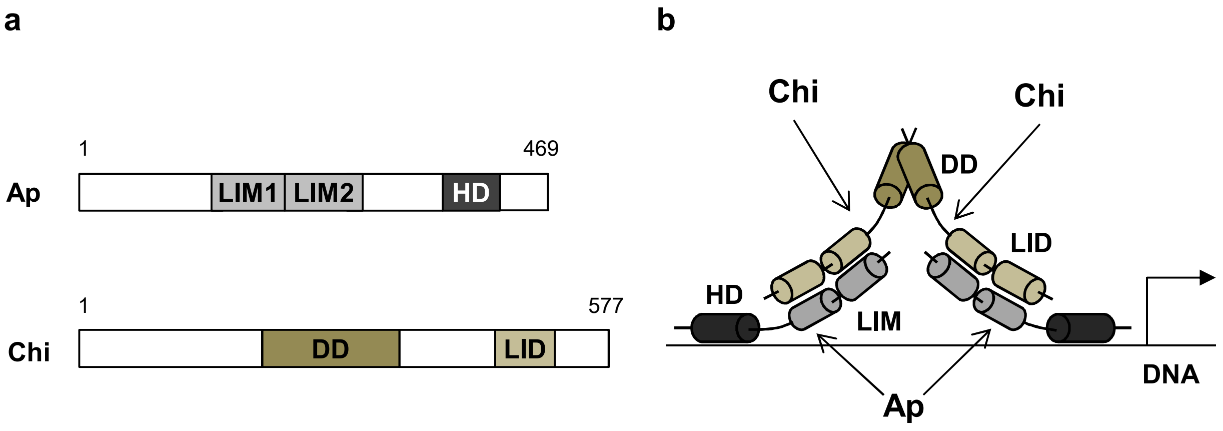

Supplementary Figure S1. Schematic representation of the Ap and Chi proteins. (a) Ap contains two LIM domains and a HD. Chi contains a dimerization domain (DD) and a LIM interaction domain (LID). (b) Ap/Chi complex.

a

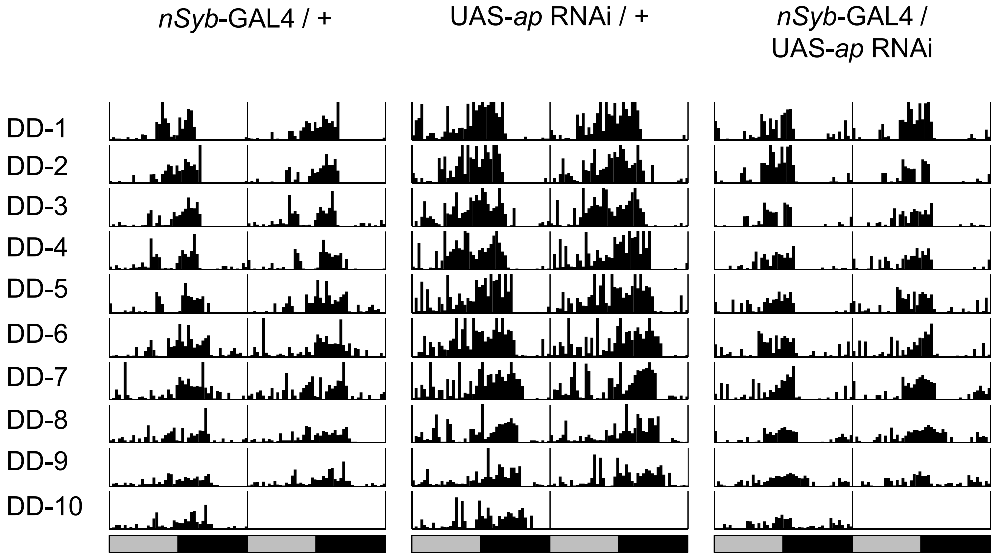

b

Flies	% rhythmic	*N*	period (h) ± SD
nSyb-GAL4/+	84.0	25	24.1 ± 0.30[a]
UAS-*ap* RANi/+	86.0	29	24.4 ± 0.19[b]
nSyb-GAL4/UAS-*ap* RNAi	100.0	32	24.0 ± 0.31[a]

Supplementary Figure S2. Panneural knockdown of *ap* does not affect behavioral rhythms in DD. (a) Double-plotted locomotor actograms of representative individual males of *nSyb*-GAL4/+, +/UAS-*ap* RNAi, and *nSyb*-GAL4/UAS-*ap* RNAi. Each actogram shows locomotor activity for 10 d of DD at 25°C. The bar under each actogram indicates subjective day (gray) and night (black). (b) Percentages of rhythmic flies and mean circadian period of DD. Periods (h) with the same superscript letters indicate that they have no significant difference.

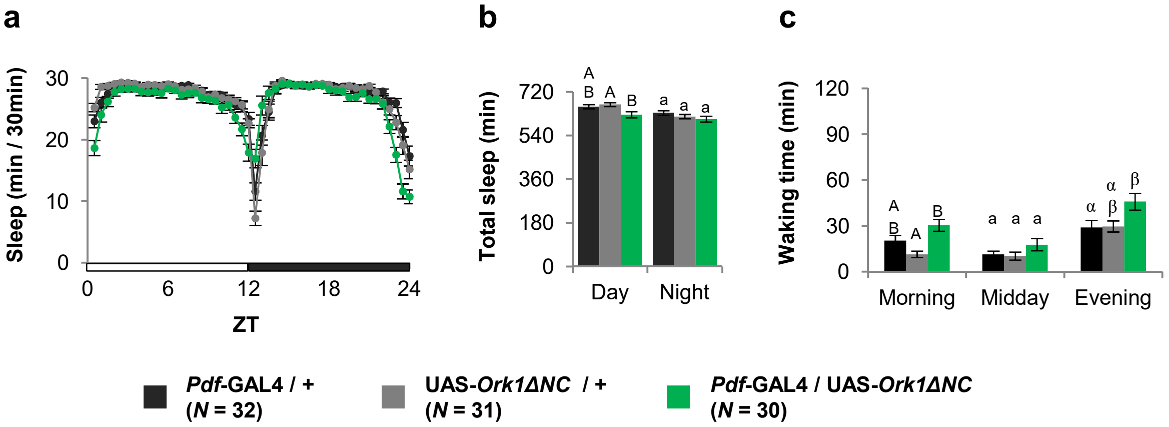

Supplementary Figure S3. Targeted expression of dOrk1ΔNC in PDF neurons. Sleep/wake parameters were analyzed using the data averaged over 3 days of LD. Error bars show S.E.M. in each figure. Bars with the same letter indicate values that are not significantly different ($P > 0.05$). (a) Daily sleep patterns of control and experimental flies. (b) Total sleep amount during day and night. (c) Waking times in the morning (ZT0-4), midday (ZT4-8), and evening (ZT8-12).

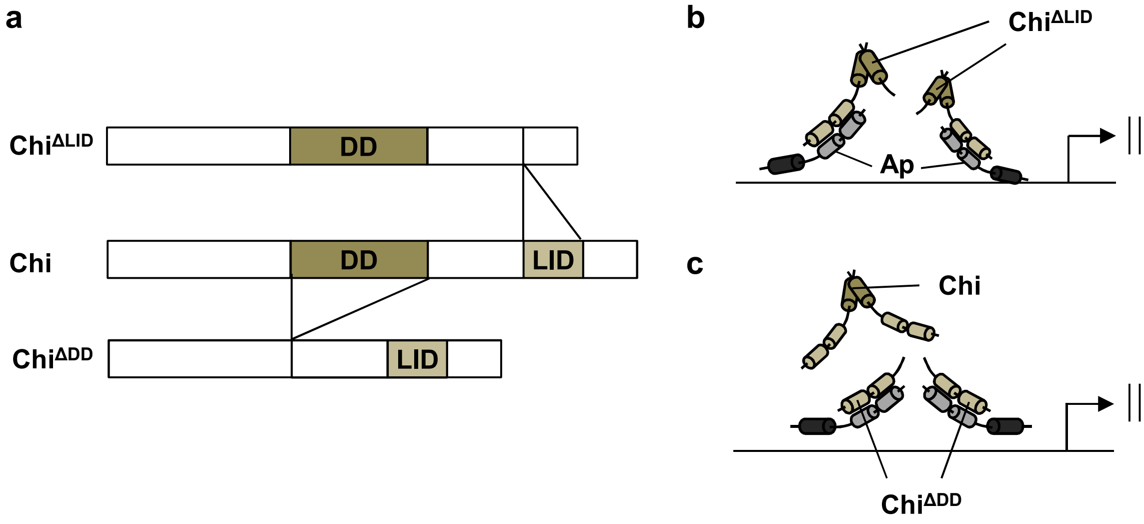

Supplementary Figure S4. Wild-type and dominant-negative forms of Chi.

(a) Schematic representation of wild-type and dominant-negative forms of Chi. (b and c) Possible model of dysfunction of Ap/Chi caused by expression of Chi$^{\Delta LID}$ and Chi$^{\Delta DD}$.

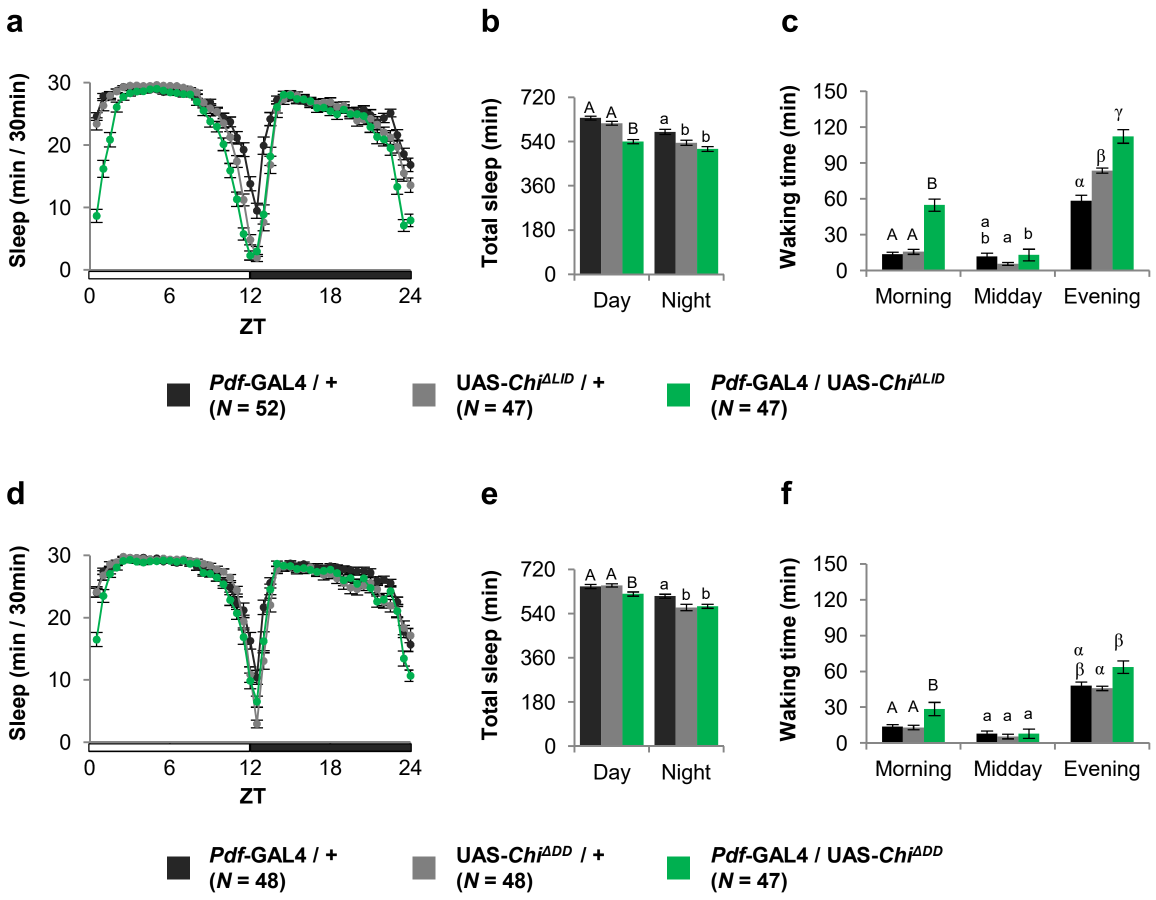

Supplementary Figure S5. Targeted expression of dominant-negative forms of Chi in PDF neurons promotes arousal. Sleep/wake parameters were analyzed using the data averaged over 3 days of LD. Error bars show S.E.M. in each figure. Bars labeled with the same letter indicate values that are not significantly different ($P > 0.05$). (a-c) *Pdf*-GAL4/UAS-$Chi^{\Delta LID}$ flies were used. (d-f) *Pdf*-GAL4/UAS-$Chi^{\Delta DD}$ flies were used. (a and d) Daily sleep patterns of control and experimental flies. (b and e) Total sleep amount during day and night. (c and f) Waking times in the morning (ZT0-4), midday (ZT4-8), and evening (ZT8-12).

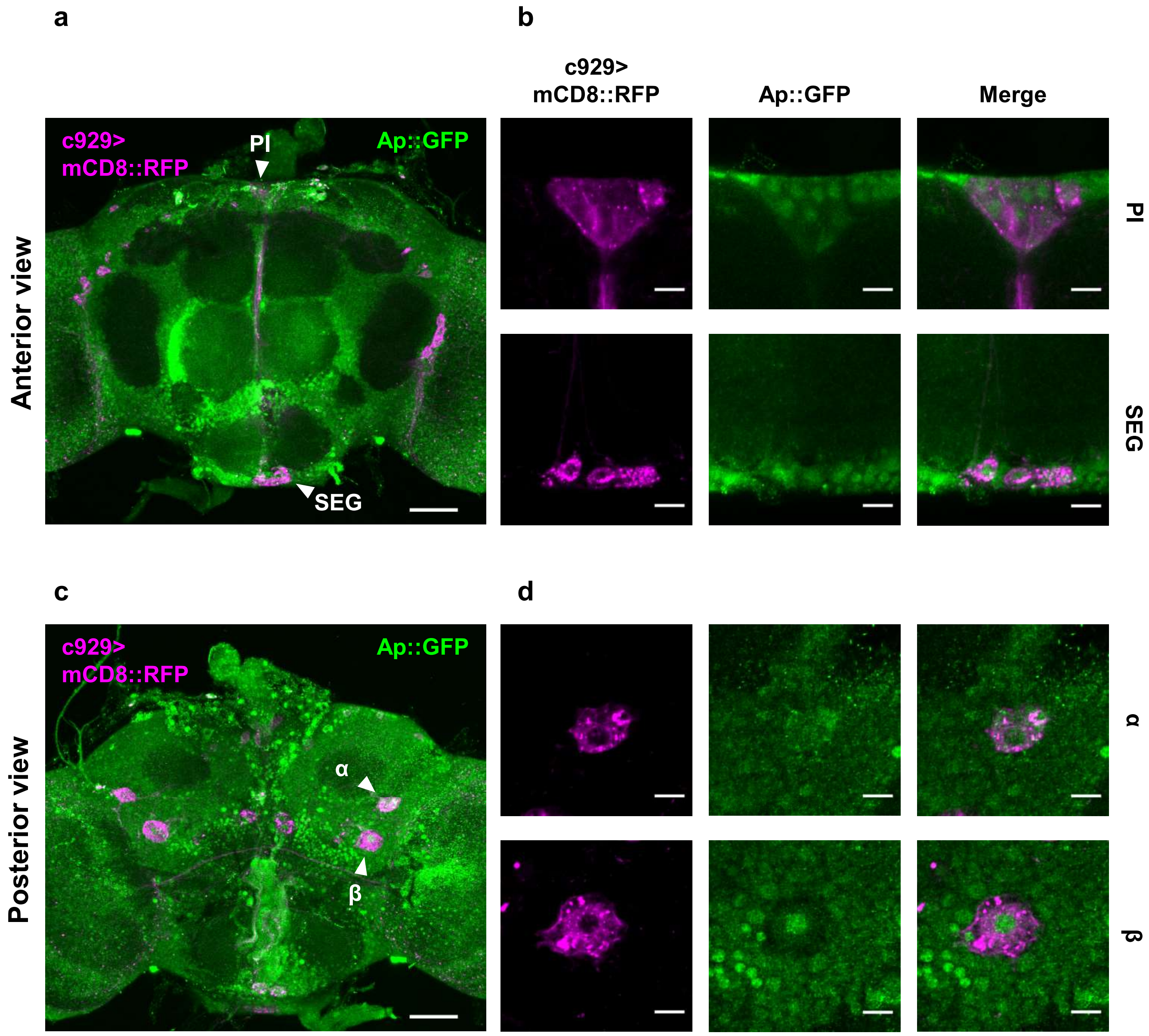

Supplementary Figure S6. Ap is expressed in c929-positive non-PDF neurons. c929-positive neurons were visualized using mCD8::RFP. c929/*ap::GFP*; UAS-IVS-*mCD8::RFP*/+ flies were used. mCD8::RFP is shown in magenta and Ap::GFP in green. Adult brains were dissected at ZT18 after the flies were entrained 7 LD cycles. (A) Stacked confocal image showing posterior view of adult brain. A scale bar represents 50 µm. (B) Partial stacked confocal image at pars intercerebralis (PI) and subesophageal zone (SEZ) levels. Scale bars represent 10 µm. (C) Stacked confocal image showing anterior view of adult brain. A scale bar represents 50 µm. (D) Confocal sectional image shown in (C) α and β. Scale bars represent 10 µm.

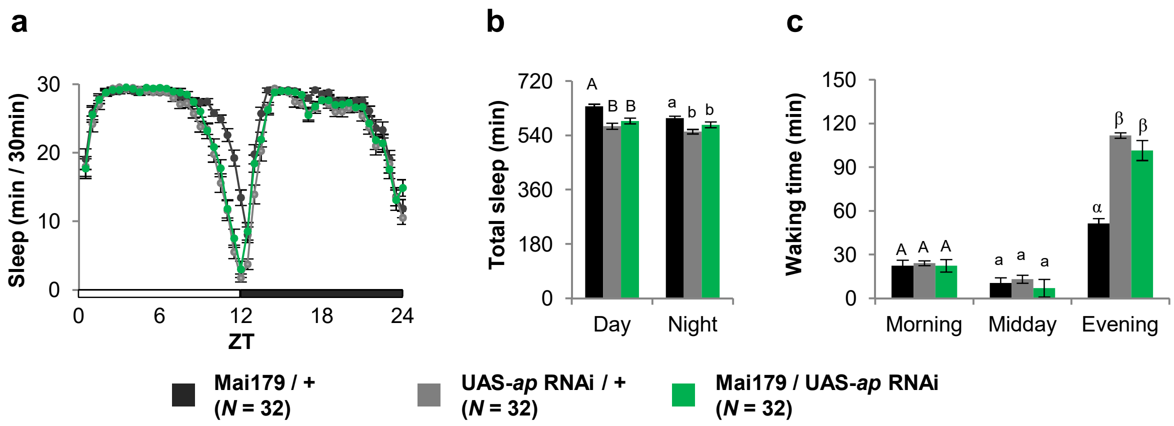

Supplementary Figure S7. ***ap* knockdown in s-LNvs does not affect the sleep/awake phenotype.** Sleep/wake parameters were analyzed using the data averaged over 3 days of LD. Error bars show S.E.M. in each figure. Bars with the same letter indicate values that are not significantly different ($P > 0.05$). (a) Daily sleep patterns of control and experimental flies. (b) Total sleep amount during day and night. (c) Waking times in the morning (ZT0-4), midday (ZT4-8), and evening (ZT8-12).

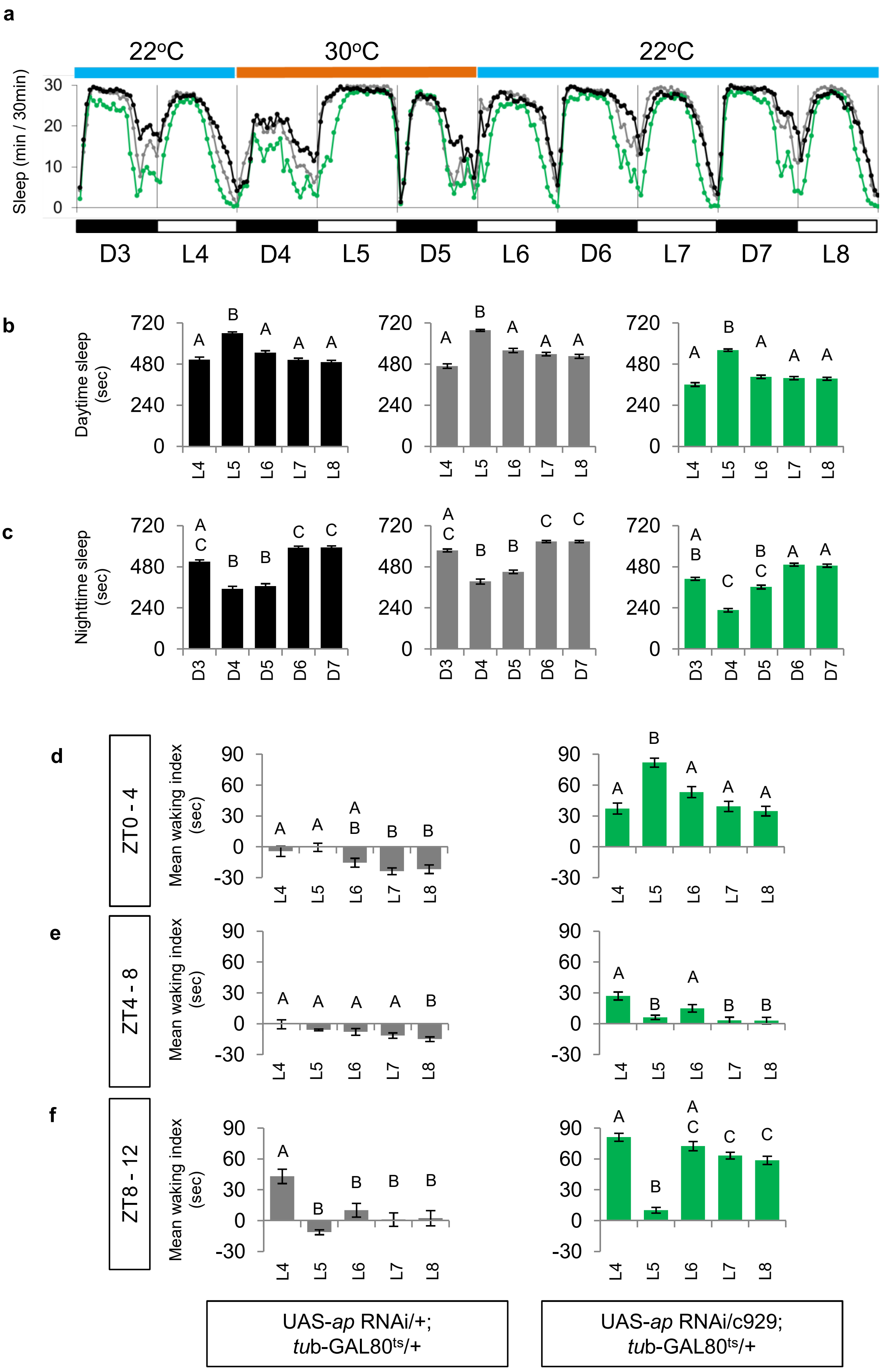

a
22°C
30°C
22°C
Sleep (min / 30min)
D3
L4
D4
L5
D5
L6
D6
L7
D7
L8
b
Daytime sleep (sec)
c
Nighttime sleep (sec)
d
ZT0 - 4
e
ZT4 - 8
f
ZT8 - 12
Mean waking index (sec)
UAS-ap RNAi/+; tub-GAL80ts/+
UAS-ap RNAi/c929; tub-GAL80ts/+

Supplementary Figure S8. Transient *ap* knockdown in l-LNvs promotes morning arousal. Using UAS-*ap* RNAi/c929; *tub*-GAL80^{ts}/+ flies (green lines and bars), we performed temperature shift experiments (22 ^{o}C–30 ^{o}C–22 ^{o}C) as shown in (a). c929/+ (black lines and bars) and UAS-*ap* RNAi/c929; *tub*-GAL80^{ts}/+ (gray lines and bars) flies were used as the control. Error bars show S.E.M. in each figure. Bars with the same letter indicate values that are not significantly different ($P > 0.05$). (a) Daily sleep patterns of control and experimental flies. (b) Total amount of daytime sleep. (c) Total amount of nighttime sleep. (d) Mean waking index in the morning (ZT0-4). (e) Mean waking index in the midday (ZT4-8). (f) Mean waking index in the evening (ZT8-12).
